# Attenuated *Salmonella* as a modulator of the cancer–immunity cycle: from innate activation to adaptive antitumor memory

**DOI:** 10.3389/fimmu.2026.1916229

**Published:** 2026-09-09

**Authors:** Sofía Chilibroste, José Alejandro Chabalgoity, María Moreno, Amy Mónaco

**Affiliations:** Unidad Académica de Desarrollo Biotecnológico, Instituto de Higiene, Facultad de Medicina, Universidad de la República, Montevideo, Uruguay

**Keywords:** antitumor response, bacterial therapy, cancer immunotherapy, cancer-immune cycle, tumor microenvironment

## Abstract

Attenuated engineered strains of *Salmonella* have emerged as promising tools in cancer immunotherapy due to their intrinsic tumor-targeting properties and their ability to reshape the tumor immune microenvironment. Beyond their direct cytotoxic effects on tumor cells*, Salmonella* profoundly modulates both innate and adaptive immune responses, impacting multiple steps of the cancer–immunity cycle. In this review, we discuss how attenuated *Salmonella* induces immunogenic tumor cell death, promotes antigen release and cross-presentation, enhances lymphocyte activation, and facilitates immune cell infiltration into the tumor microenvironment. We also address its role in controlling metastatic disease and the induction of long-term adaptive immune memory. Furthermore, we highlight unresolved questions regarding the contribution of immune activation, tolerance, and trained immunity, which may underlie the transient nature of antitumor effects observed in some models. Finally, we examine the translational hurdles that have limited clinical efficacy—such as the balance between attenuation and potency, endotoxin tolerance, and preexisting immunity—and outline emerging engineering strategies to overcome them. Understanding these mechanisms is essential for optimizing *Salmonella*-based strategies and their integration with current immunotherapies.

## Introduction

The development of effective anticancer immunotherapies has transformed cancer treatment; however, durable clinical responses remain limited to a subset of patients (1). Tumor immune evasion and the inefficient priming of antitumor immune responses continue to challenge therapeutic success (2). Strategies capable of simultaneously targeting tumor cells and reprogramming the immune system have the potential to overcome these limitations.

**Figure 1:**
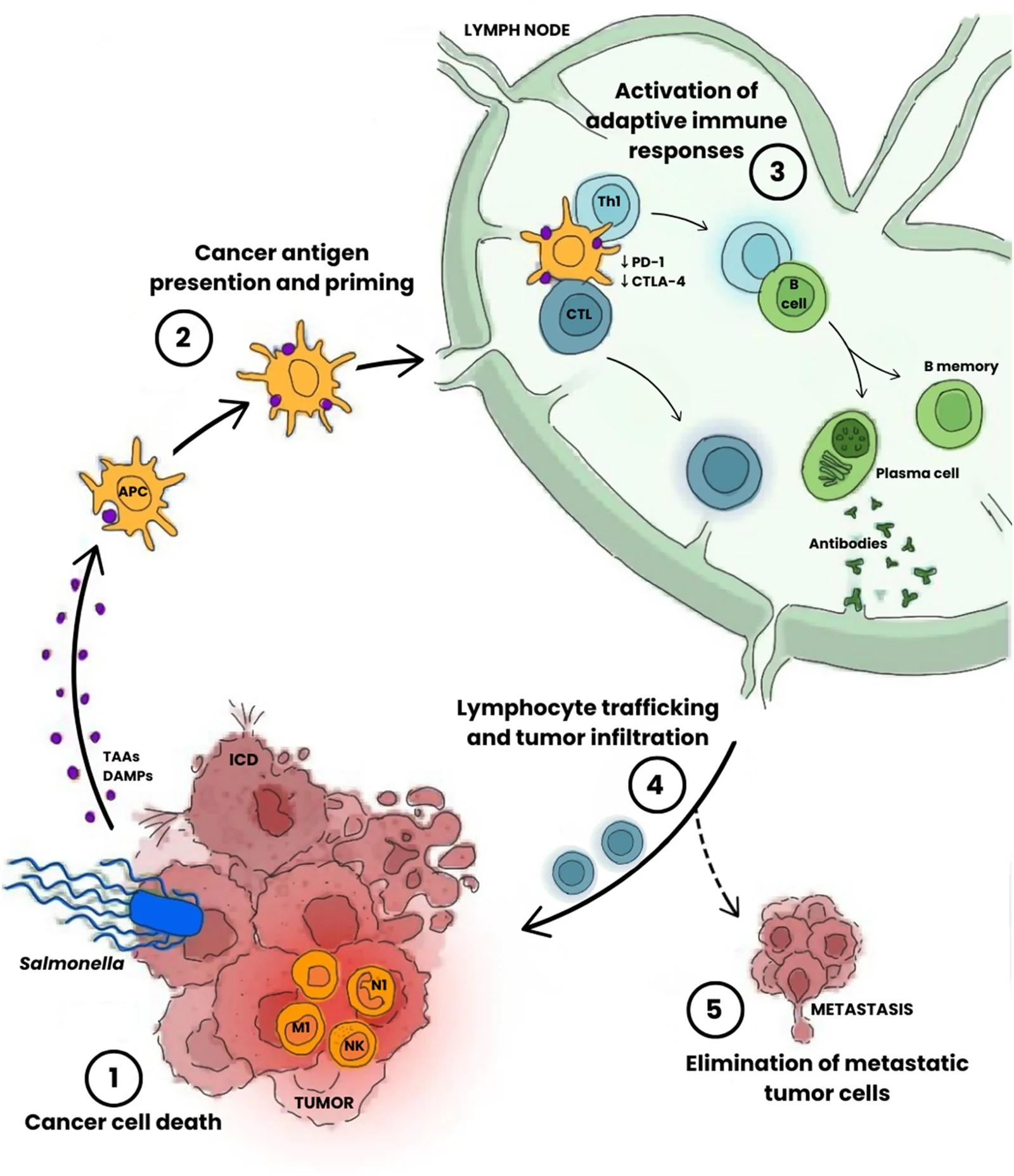
Impact of *Salmonella* on the cancer–immunity cycle and tumor immune reprogramming. Attenuated *Salmonella* initiates a multistep remodeling of the tumor immune microenvironment that enhances antitumor immunity, depicted as the cancer-immunity cycle (14). In our interpretation of this cycle: (1) within the tumor site, bacterial colonization promotes direct tumor cell killing through different pathways, which lead to immunogenic cell death (ICD) and activation of inflammatory pathways given the release of damage-associated molecular patterns (DAMPs), together with tumor-associated antigens (TAAs). This inflammatory context favors the recruitment and polarization of innate immune effectors toward an antitumor M1-like phenotype, while simultaneously counteracting tumor immune evasion mechanisms and reducing pro-tumoral angiogenic signals. (2) TAAs are then captured by antigen-presenting cells (APCs), which migrate to tumor-draining lymph nodes (TDLNs), where *Salmonella*-driven proinflammatory state facilitates antigen transfer and cross-presentation on major histocompatibility complex (MHC) class I molecules. In parallel, inhibitory immune checkpoints such as Programmed Death-1 (PD-1) and Cytotoxic T-lymphocyte-associated protein 4 (CTLA-4) are downregulated, favoring productive T cell priming. (3) This leads to the activation of tumor-specific adaptive immune responses, involving B cells, T helper 1 (Th1)-polarized CD4^+^ T cells, cytotoxic CD8^+^ T lymphocytes, and the establishment of long-term immunological memory. (4) Activated effector lymphocytes subsequently traffic back to the tumor bed, where enhanced infiltration and cytotoxic activity contribute to tumor control. (5) Additionally, systemic antitumor immunity supports the elimination of metastatic tumor cells, extending immune surveillance beyond the primary tumor site.

In this regard, microorganism-based immunotherapies have emerged as a powerful strategy to couple direct tumor targeting with immune activation (3), as exemplified by the clinical success of the oncolytic herpesvirus talimogene laherparepvec (T-VEC) in melanoma (4) or the use of Bacillus Calmette-Guérin (BCG) as treatment of non-muscle invasive bladder cancer (5, 6). Other attenuated microorganisms, such as different serovars of the bacteria *Salmonella enterica*, have also been explored as anticancer agents due to their preferential accumulation within tumors, driven by hypoxia, necrosis, and aberrant vasculature, among other characteristics (7). While various *Salmonella* species and serovars exist, the vast majority of preclinical and clinical studies evaluating bacterial cancer immunotherapy utilize attenuated strains of non-typhoidal *Salmonella enterica* serovar Typhimurium (e.g., VNP20009, A1-R, YB-1 mutants) (8). These strains are genetically engineered through specific attenuation strategies, including metabolic auxotrophies (e.g., purI, aroA, aroC deletions), disruption of ion transport systems such as the ZnuABC zinc transporter, or lipid A modification (e.g., msbB deletion to diminish septic toxicity), balancing safety with preserved immunostimulatory capacity (9–12). Although other non-typhoidal serovars such as *S*. Enteritidis or attenuated typhoidal *S*. Typhi strains have been explored to a lesser extent, attenuated *S*. Typhimurium mutants share core mechanisms of tumor targeting, pattern recognition receptor activation, and immune microenvironment remodeling. Throughout this review, unless specified otherwise, mechanistic descriptions derive primarily from attenuated *S*. Typhimurium models, which represent the primary benchmark in the field. Even though early approaches focused primarily on the direct cytotoxic effects of bacterial colonization, accumulating evidence indicates that the antitumor efficacy of attenuated *Salmonella* largely depends on its capacity to modulate host immunity, functioning not merely as a tumoricidal agent or delivery vector, but as a potent immune modulator that activates innate immune pathways, reshapes the tumor microenvironment (TME), and promotes adaptive antitumor responses (13). These effects position *Salmonella* as a unique regulator of the cancer–immunity cycle, influencing tumor antigen release, antigen presentation, lymphocyte priming and activation, immune cell trafficking and infiltration on TME, as well as T cell recognition of tumor cell and elimination (**Figure 1**).

Many existing reviews have discussed tumor targeting, bacterial engineering, immune modulation of the tumor microenvironment, and combination therapies. In contrast, this review focuses on mapping *Salmonella*-mediated impact across the different stages of the cancer-immunity cycle (14), emphasizing the underlying immune-mediated mechanisms. While this paradigm, as originally proposed by Chen and Mellman, centers on the priming and execution of CD8+ T cell-mediated responses, the multifaceted nature of *Salmonella* as an immunomodulator suggests a broader orchestration of the cycle. Therefore, we propose a reinterpretation of this framework (**Figure 1**), arguing that in the context of bacterium-based therapies, the cycle’s progression does not rely solely on the adaptive arm; and instead, it integrates potent innate effector mechanisms—such as Natural Killer (NK) cell activation, macrophage reprogramming, and trained immunity—which can also drive tumor control and shape long-term immune memory. In this way, *Salmonella*-induced inflammation initiates a cascade of immune events that bridge innate and adaptive immunity, ultimately leading to antitumor effector responses. We further discuss how these processes converge to generate antitumor immunity, while addressing the potential involvement of immune tolerance and innate immune memory in shaping therapeutic outcomes.

## Direct tumor cell killing and induction of immunogenic cell death

**Figure 2:**
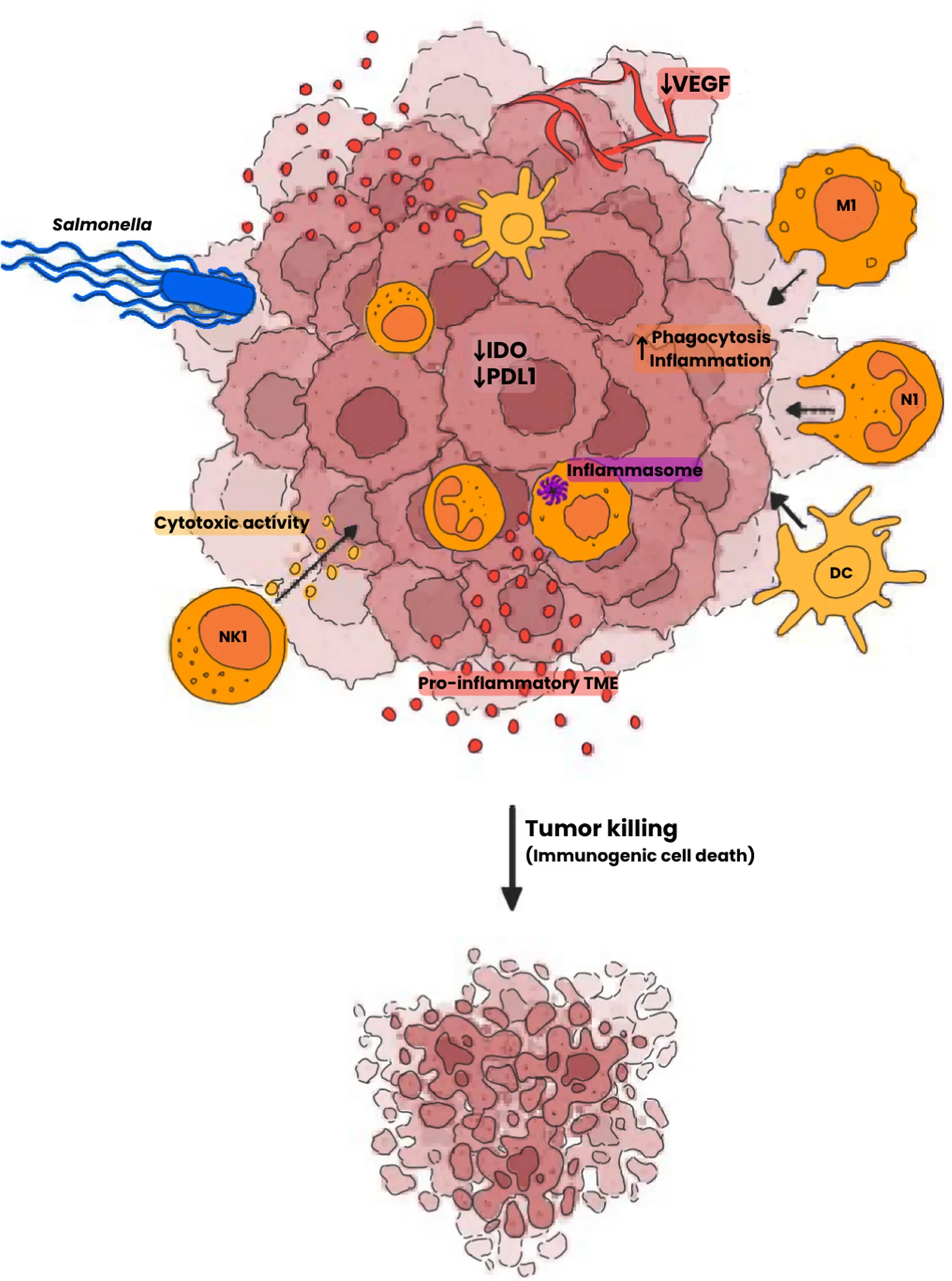
*Salmonella*-driven inflammatory remodeling of the tumor microenvironment promotes local tumor control. *Salmonella* induces a pro-inflammatory reprogramming of the tumor microenvironment that favors antitumor immunity. Bacterial presence within tumors promotes immunogenic tumor cell death and inflammasome-associated inflammation, leading to the recruitment and activation of innate immune effectors with antitumor functions. This inflammatory milieu supports cytotoxic activity by natural killer (NK) cells and other immune effectors, enhances phagocytosis of tumor cells by myeloid populations, and counteracts tumor-intrinsic immune resistance mechanisms, including immunosuppressive pathways and pro-angiogenic signals.

One of the earliest and most extensively described effects of attenuated *Salmonella* in initiating the very first step of the cancer–immunity cycle, is its ability to induce direct tumor cell death, where preferential tumor colonization serves as a crucial prerequisite for both antigen release and subsequent antitumor efficacy. Multiple preclinical studies have demonstrated that preferential bacterial accumulation within tumor tissue is required to induce local inflammation, tumor cell death, and early remodeling of the TME, whereas strains defective in tumor colonization or non-viable bacteria fail to elicit these responses (15–19). Although attenuated *Salmonella* has been shown to invade, survive, and in some contexts replicate within tumor cells *in vitro* and *in vivo* (20–23), intracellular localization within tumor cells remains context-dependent. While some studies support a role for intracellular bacterial infection in inducing tumor cell stress and immune activation, others indicate that comparable inflammatory and immunomodulatory effects can be achieved in the absence of direct intracellular tumor cell infection (24), potentially through extracellular pattern recognition receptor engagement or through infection and activation of tumor-infiltrating immune cells, particularly myeloid populations (25, 26). These non-mutually exclusive mechanisms likely reflect differences in TME, bacterial characteristics, and host immune composition. Following preferential colonization of the tumor tissue, *Salmonella* can indirectly promote tumor cell killing through metabolic competition (27). Recent evidence indicates that bacterial presence depletes key substrates like asparagine, disrupting tumor metabolic homeostasis which leads to both arrest of proliferation and cell death (28). Additionally, the induction of cellular stress pathways also leads to cell death that frequently exhibits features of immunogenic cell death (ICD) (29, 30), converting dying tumor cells into a source of danger signals capable of activating the immune system (**Figure 2**). ICD is characterized by the release of damage-associated molecular patterns (DAMPs) and pro-inflammatory mediators, such as high mobility group box 1 (HMGB1) or adenosine triphosphate (ATP) that facilitate immune cell recruitment and activation (31). HMGB1 has been reported to be actively released by *Salmonella*-infected tumor cells (30) and macrophages in a caspase-1–dependent manner (32), further contributing to the local inflammatory response. Given that extracellular HMGB1 can also function as a toll-like receptor 4 (TLR4) agonist (33), we hypothesize that its release may represent an additional mechanism by which *Salmonella* amplifies innate immune activation within the TME. In the context of *Salmonella*-based therapies, bacterial components such as lipopolysaccharide (LPS) and flagellin also engage pattern recognition receptors, including Toll-like receptors and cytosolic sensors, amplifying inflammatory signaling within the TME (30, 34–36). This inflammatory milieu promotes the activation of nuclear factor kappa-light-chain-enhancer of activated B cells (NFкB) and inflammasome pathways and the secretion of cytokines as interleukin 1β (IL-1β), interferon-γ (IFN-γ), interleukin 6 (IL-6) and tumor necrosis factor (TNF), among many others (30, 37–40), which favor antitumor immunity. Engineered strains have also been developed to optimize the regulated secretion of additional therapeutic proteins directly into the tumor, further enhancing cytotoxicity and targeted antitumor activity, leading to increased tumor necrosis and reduced mitotic activity (41). Of note, besides targeting tumor cells and immune effectors, attenuated *Salmonella* strains—such as *S*. Typhimurium ΔznuABC—have also been reported to modulate non-immune stromal components within the TME, including cancer-associated fibroblasts, at least in an *in vitro* infection scenario (42).

Concomitantly, *Salmonella* infection contributes to the recruitment and polarization of innate immune effectors toward an antitumor phenotype. Tumor-associated macrophages (TAMs), which are often skewed toward an immunosuppressive state, can be reprogrammed toward a pro-inflammatory, M1-like phenotype characterized by enhanced phagocytic activity and cytokine production (30, 43). Neutrophils, NK cells and dendritic cells (DCs) are also recruited to the tumor site, further reinforcing local immune activation (11, 37, 39).

In addition to shaping immune cell composition, *Salmonella* has been reported to interfere with key tumor-intrinsic mechanisms of immune resistance. Reduced expression of immunosuppressive molecules such as indoleamine 2,3-dioxygenase (IDO), programmed death-ligand 1 (PD-L1) and lymphocyte-activation gene 3 (LAG-3) has been observed in tumors following *Salmonella* treatment, potentially lowering the threshold for effective immune-mediated tumor cell killing (44–46). Moreover, the downregulation of vascular endothelial growth factor (VEGF) and alterations in tumor vasculature may restrict tumor growth (47) while simultaneously facilitating immune cell infiltration.

Together, these effects position *Salmonella*-induced tumor cell death not merely as a cytotoxic event, but as a critical initiating step in the cancer–immunity cycle. By coupling direct tumor destruction with inflammation and immune activation, attenuated *Salmonella* creates the conditions necessary for efficient tumor-associated antigen (TAA) release and presentation in Step 2, and the subsequent priming of adaptive antitumor responses.

## Tumor antigen release and cross-presentation

The induction of tumor cell death by attenuated *Salmonella* allows the release and subsequent capture of TAAs that leads to the second step of the cancer–immunity cycle. This immunogenic event is accompanied by inflammatory signals that promote the recruitment and activation of professional antigen-presenting cells (APCs), particularly dendritic cells, thereby enhancing antigen uptake and processing (29).

Within the TME, DCs and other phagocytic cells internalize tumor-derived antigens released (29). Concurrent exposure to bacterial pathogen-associated molecular patterns provides strong maturation signals to these APCs, promoting upregulation of co-stimulatory molecules and the production of pro-inflammatory cytokines (48, 49). As an example of the relevance of pattern recognition receptors (PRRs) in cancer, a study showed that TLR4 deficient mice fail to successfully maturate and activate DCs, as well as produce IFN-γ (50). This dual exposure to tumor antigens and microbial signals is a key feature distinguishing *Salmonella*-based approaches from other tumor-debulking strategies. Consistent with this concept, HMGB1 released from dying tumor cells in the context of *Salmonella*-induced tumor disruption may further support antigen presentation. HMGB1–TLR4 interactions have been shown to be critical for dendritic cell maturation and efficient processing of TAAs, thereby enabling effective priming of tumor-specific T cells (51).

A notable mechanism that may further enhance antigen presentation in this context is the modulation of gap junction proteins, such as connexin-43 (Cx43). Increased expression of Cx43 has been associated with improved intercellular communication between tumor cells and APCs, facilitating the transfer of tumor antigens and favoring cross-presentation via major histocompatibility complex class I (MHC I). This process is essential for the priming of cytotoxic T lymphocytes (CTLs) capable of recognizing and eliminating tumor cells (52).

Beyond antigen processing, and bridging toward effective T cell priming, *Salmonella*-induced inflammation also influences immune checkpoint pathways that regulate T cell activation. Interestingly, *Salmonella*-induced dose-dependent Cx43 upregulation in a melanoma cell line was shown to reduce IDO expression, and conditioned medium from treated tumor cells enhanced T cell proliferation, indicating that *Salmonella* reduces both the expression and function of IDO in tumor cells through a Cx43 dependent mechanism (53). In addition, HMGB1 has been shown to modulate regulatory T cell (Treg) function through TLR4 signaling. *In vitro* studies demonstrated that HMGB1 exposure attenuates the suppressive activity of CD4^+^CD25^+^ Tregs, weakening their ability to inhibit conventional T cell proliferation and altering their cytokine expression profile via NF-κB–dependent mechanisms (54). Given that *Salmonella* infection promotes HMGB1 release by multiple cell types within the TME, it is plausible to infer that *Salmonella*-driven inflammation may also contribute to the attenuation of Treg–mediated immunosuppression *in vivo*.

Following antigen uptake and maturation, APCs migrate to tumor-draining lymph nodes (TDLNs), where they present tumor-derived peptides to naïve T cells. This step represents a critical amplification point within the cancer–immunity cycle, as it links localized tumor inflammation to systemic adaptive immune activation. Along with APCs, increased T cells numbers (due to infiltration or expansion) into TDLNs have also been shown in different preclinical models (39, 40). Importantly, in the presence of *Salmonella*, TDLN lymphocytes decreased CTLA-4 and PD-1 expression, indicating T cell function reinvigoration (39). In this framework, attenuated *Salmonella* acts not only as a trigger of antigen release, but also as a facilitator of efficient antigen presentation and T cell priming, thereby driving the transition toward T cell priming and downstream effector responses. In parallel, *Salmonella* also impacts on the peripheral immune system, demonstrated by an increase in the percentage of splenic CD4+ T cells was confirmed in a preclinical colon carcinoma model (44), further amplifying adaptive immune activation in Step 3 of the cycle (**Figure 1**).

## Activation of adaptive immune responses

**Figure 3:**
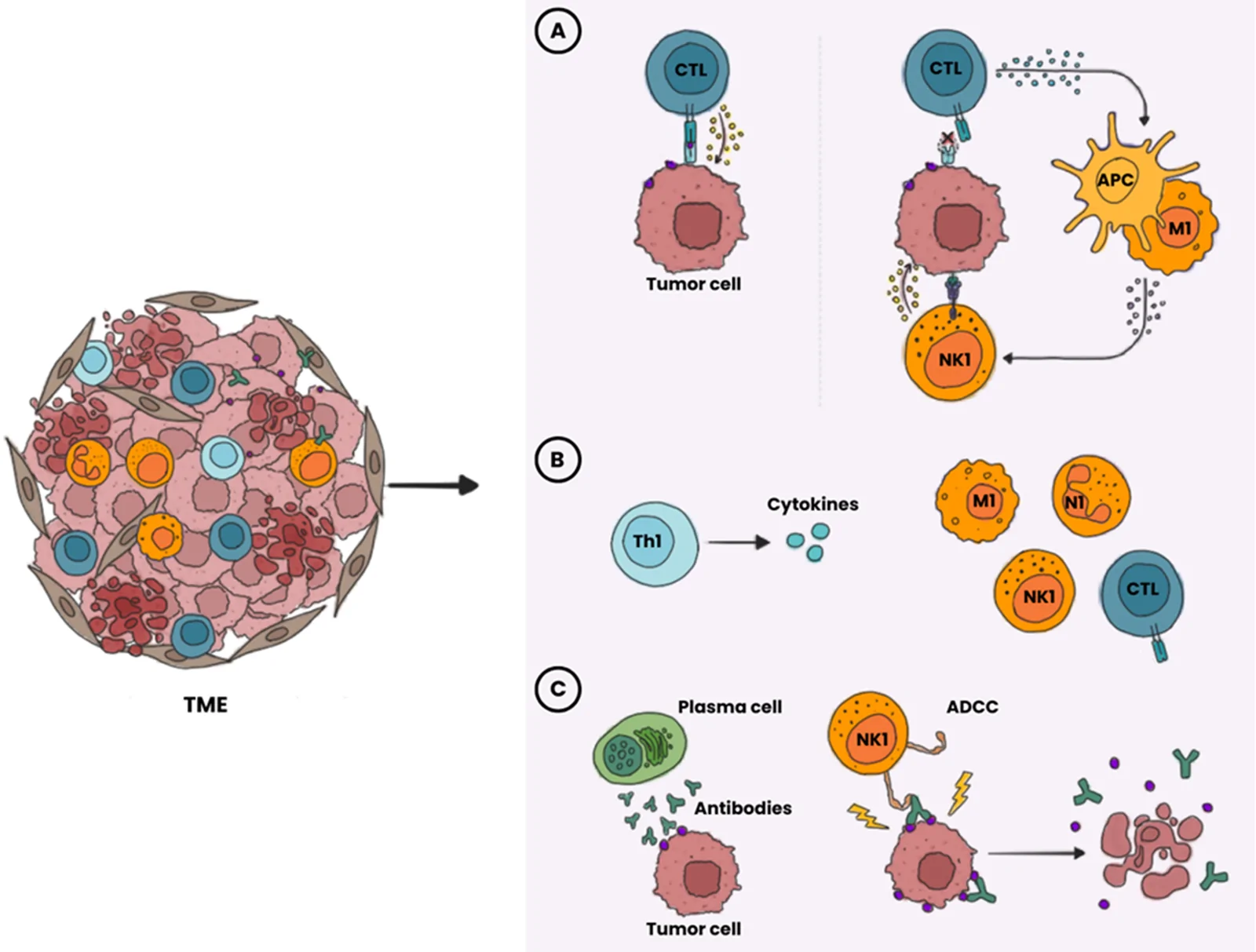
Immune-mediated tumor cell elimination upon cancer-immunity cycle modulation by *Salmonella*. **(A)** Tumor antigen–specific cytotoxic T lymphocytes (CTLs) mediate tumor control through both direct and indirect mechanisms. CTLs directly recognize and kill malignant cells, and additionally promote antitumor immunity by modulating antigen-presenting cells (APCs) and macrophages, which in turn enhance natural killer (NK) cell activation and cytotoxic function within the tumor microenvironment (TME). **(B)** CD4^+^ Th1 cells reinforce antitumor immunity by promoting a pro-inflammatory milieu that supports the activation and function of immune effector cells, such as NK cells, M1 macrophages, N1 neutrophils and CTLs. **(C)** Antibody-dependent cellular cytotoxicity (ADCC) contributes to tumor cell elimination within the remodeled TME, primarily through the engagement of Fc receptor–expressing effector cells such as NK cells.

Representing the third step of the cancer–immunity cycle (**Figure 1**), efficient antigen presentation driven by attenuated *Salmonella* culminates in the robust priming and activation of adaptive immune responses, encompassing both B and T cell lineages. The inflammatory environment generated by bacterial sensing and tumor antigen release strongly favors the differentiation and expansion of effector lymphocyte populations with antitumor activity. In particular, *Salmonella*-based strategies have been associated with a pronounced skewing toward type 1 immune responses, characterized by the activation of CTLs and T helper 1 (Th1) cells (45, 55, 56) (**Figure 3**). In this scenario, the induction of effector CD8+ T cells by recombinant strains has been widely documented through complementary methodologies (57–59).

CD8^+^ CTLs represent a central effector population in *Salmonella*-induced antitumor immunity. Upon priming by cross-presenting dendritic cells, CTLs acquire cytotoxic functions that enable direct recognition and killing of tumor cells through perforin- and granzyme-mediated mechanisms (37). Notably, this cytotoxic axis can be further potentiated through genetic engineering of *Salmonella* strains. For example, *Salmonella* modified to express interleukin 21 (IL-21) enhanced the intratumoral expression of both perforin and granzymes and resulted in superior antitumor efficacy compared to either unmodified *Salmonella* or IL-21 administration alone, indicating that bacterial delivery of immunostimulatory cytokines can amplify CTL effector function within the TME (22). The presence of bacterial-derived inflammatory signals during priming also enhances CTL functionality and promotes the production of effector cytokines such IFN-γ (39), which further amplifies antitumor immune responses and contributes to the remodeling of the TME. In line with this, whereas IFN-γ is dispensable for *Salmonella*-mediated control of primary melanoma growth, it showed essential for exerting protection against disease spread (60).

Th1 CD4^+^ T cells play a complementary role by providing help to CTLs and shaping the overall immune landscape. Th1-derived cytokines, including IFN-γ and TNF, support macrophage activation, promote antigen presentation, and reinforce cytotoxic effector functions (61). In addition, Th1 responses counteract immunosuppressive pathways within the TME, favoring sustained immune pressure against tumor cells (2, 62). Interestingly, tumor-specific CD4+ T cells have been shown to exert other functions beyond helping CD8+ T cells. In that regard, a study using single-cell sequencing on localized muscle-invasive bladder patient samples defined multiple cytotoxic CD4+ T cell states that effectively kill tumor cells directly through granzymes, other granules and perforin (63). Although direct cytotoxic activity of CD4^+^ T cells against tumor cells has not been conclusively demonstrated in the *Salmonella*-treatment setting, we speculate that the inflammatory and effector programs induced by bacteria create conditions compatible with broader CD4^+^ effector functions beyond classical helper role.

Although less extensively characterized, B cell responses may also participate in *Salmonella*-driven antitumor immunity. Antibody production against TAAs can facilitate opsonization and antibody-dependent cellular cytotoxicity (ADCC), while B cells may additionally function as antigen-presenting cells, contributing to T cell activation and memory formation. Approaches administering different *Salmonella* species by diverse routes have revealed B cells increase in both tumors and TDLN as soon as 7 days post injection (40, 43). Additionally, significant serum antitumor immunoglobulin G (IgG) levels have been detected post *Salmonella* treatment of different cancer preclinical models (11, 23). ADCC further expands the repertoire of effector mechanisms, linking humoral responses to innate cytotoxicity and reinforcing tumor cell elimination. Innate lymphocytes, particularly NK cells, contribute to this effector phase of the immune response elicited by *Salmonella* (16, 64). Besides, NK cells can directly eliminate tumor cells that downregulate MHC class I expression, a common immune escape mechanism, and can cooperate with adaptive immune cells through cytokine-mediated crosstalk (reviewed in 65). In this regard, partial NK cell depletion has shown to abrogate the early tumor reduction observed soon after *Salmonella* treatment (39).

Through the activation of CTLs and Th1 cells, together with the engagement of NK cells and antibody-mediated effector pathways, attenuated *Salmonella* seems to promote a multifaceted adaptive immune response capable of targeting heterogeneous tumor cell populations. This integrated immune activation represents a key step in the cancer–immunity cycle and provides a mechanistic basis for the systemic antitumor effects observed in multiple experimental models, while also contributing to the amplification of the cycle through sustained tumor killing and continued antigen release. Importantly, once these immune cells are primed in secondary lymphoid organs, they must successfully reach the tumors, setting the stage for Step 4 of the cycle: lymphocyte trafficking and tumor infiltration.

## Lymphocyte trafficking, tumor infiltration, and control of metastatic disease

The activation of adaptive immune responses following attenuated *Salmonella* treatment must be accompanied by efficient immune cell trafficking and infiltration into tumor tissues to achieve effective tumor control, allowing the next steps of the cancer-immunity cycle. One of the critical downstream consequences of *Salmonella*-induced immune activation is the remodeling of the TME toward a pro-inflammatory state that favors immune cell recruitment and retention. Increased production of inflammatory cytokines and chemokines, such as C-X-C motif chemokine ligands 9 and 10 (CXCL9 and CXCL10, respectively), together with changes in stromal and vascular compartments, promotes the infiltration of adaptive immune cells into tumor tissue (11, 39, 66). In addition, inflammation-driven upregulation of adhesion molecules, such as intercellular adhesion molecule 1 (ICAM-1), within the TME can contribute to the retention of activated lymphocytes at tumor sites, thereby sustaining local antitumor immune responses (66, 67). Although direct evidence that *Salmonella* increases ICAM-1 expression in tumors remains limited, pro-inflammatory cytokines induced during *Salmonella* colonization, such as TNF and IFN-γ, are well-established regulators of adhesion molecule expression (68). Moreover, LPS has been shown to upregulate ICAM-1 expression in cancer cells (69), and ICAM-1 expression has been documented in inflamed tissues during *Salmonella* infection, including in and around granulomas in infected mice (70). Taken together, these findings suggest that *Salmonella*-driven inflammation is associated with adhesion molecule expression *in vivo*, which in turn promotes lymphocyte accumulation in tumors.

Strategies forcing bacteria to specifically express chemokines as chemokine C-C motif ligand 21 (CCL21) can further improve antitumor effect through T lymphocytes (71). Enhanced infiltration of CD8^+^ cytotoxic T lymphocytes, Th1 cells, and NK cells into the TME has been reported across multiple experimental models, correlating with improved tumor control (11, 39, 66). This infiltration is further supported by vascular remodeling within the tumor, potentially driven by reduced VEGF levels and altered endothelial activation, which may improve immune cell access to tumor tissue (47).

Once within the TME, infiltrating lymphocytes exert direct cytotoxic effects on tumor cells and contribute to the maintenance of a pro-inflammatory milieu that sustains antitumor immunity. IFN-γ produced by CTLs and Th1 cells enhances antigen presentation by tumor and stromal cells, while also inhibiting tumor cell proliferation and survival. In parallel, macrophages and other innate immune cells activated by *Salmonella* cooperate with lymphocytes to amplify tumor cell killing and limit immune escape mechanisms (**Figure 3**). Crucially, this wave of tumor destruction completes the cancer–immunity cycle and initiates a self-sustaining feedback loop, releasing fresh tumor antigens to continually re-prime adaptive immunity. Recent work has highlighted that the TME can also support anti-tumor immunity through the formation of peri-tumoral lymphoid aggregates, known as tertiary lymphoid structures (TLSs). These structures have been associated with enhanced T-cell responses and improved clinical outcomes. Accordingly, the composition and frequency of TLSs are emerging as important features associated with responsiveness to immunotherapy (14). However, the potential impact of *Salmonella*-based therapies on this emerging component of the cancer–immunity cycle remains unknown. This limitation is partly explained by the tumor models commonly used in these studies, which typically do not develop tertiary lymphoid structures.

Using genetically modified mouse models with defined immune deficiencies, early studies provided important insights into the immune mechanisms underlying *Salmonella*-mediated antitumor effects. In particular, Luo et al. demonstrated that the antitumor activity of the attenuated *Salmonella* strain VNP20009 did not strictly depend on the presence of T and B lymphocytes, as significant tumor growth inhibition was observed even in immunodeficient mice (72). Similarly, tumor reduction has been reported in models lacking CD4^+^ T cells following *Salmonella* treatment (73), further supporting the notion that innate immune activation and direct bacterial effects play a dominant role in controlling primary tumor growth.

**Figure 4:**
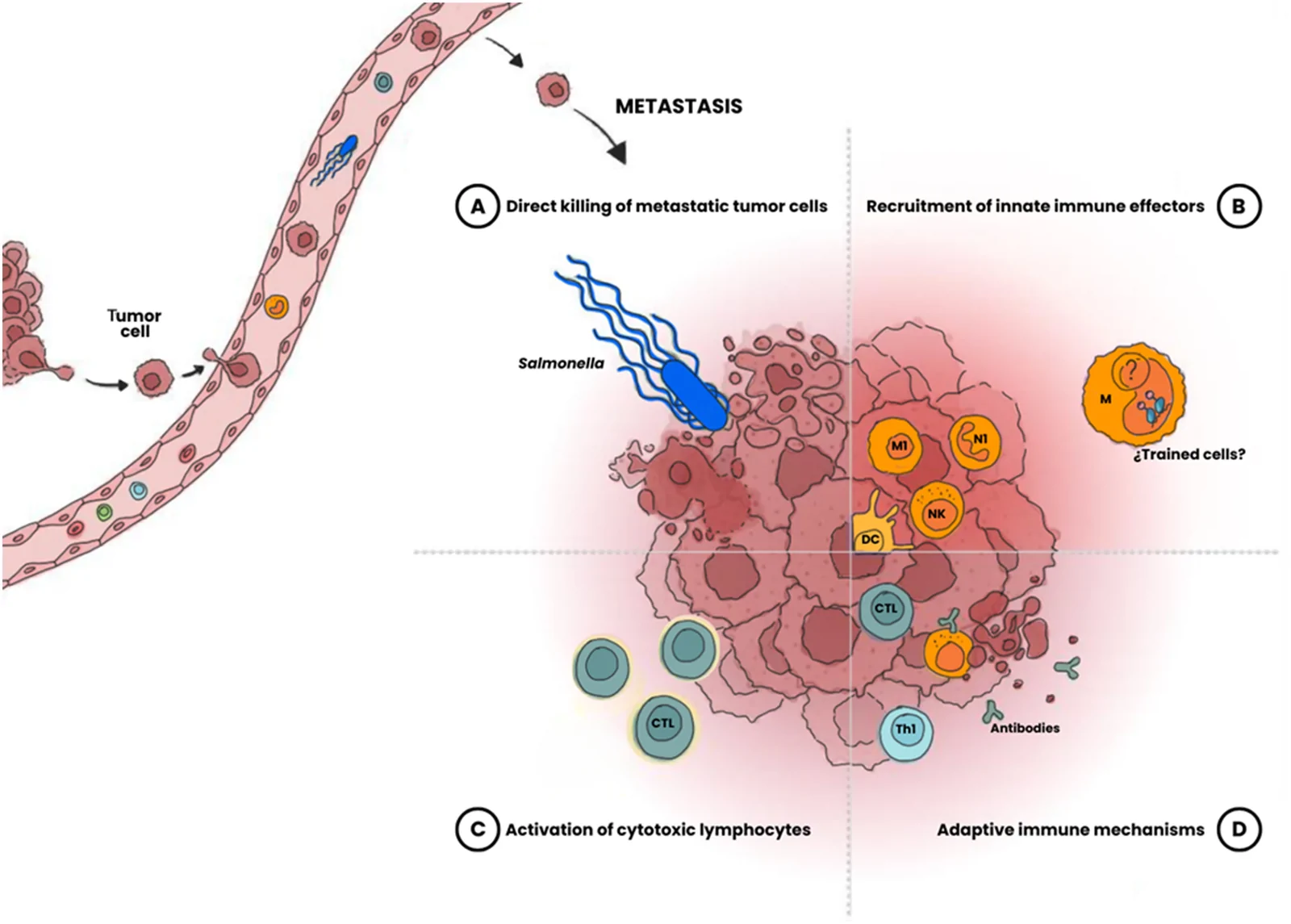
Control of metastases by *Salmonella*-based immunotherapy. **(A)** Persistence of attenuated *Salmonella* in circulation or administration of booster doses promotes colonization of metastatic niches, enabling direct killing of metastatic tumor cells. **(B)** Recruitment and polarization of innate immune effectors toward an antitumoral phenotype, together with the potential engagement of trained innate cells primed by previous treatments. **(C)** Activation of memory cytotoxic T lymphocytes (CTLs) capable of recognizing tumor-associated antigens (TAAs) and mediate tumor cell killing. **(D)** Engagement of additional antitumoral adaptive immune mechanisms contributes to systemic immune surveillance and metastatic control.

Together, these findings highlight that, at the level of the primary tumor, *Salmonella*-induced antitumor activity can be largely driven by innate immune mechanisms, local inflammatory responses, and the direct tumor-targeting properties of the bacterium. Importantly, the systemic nature of the immune response elicited by attenuated *Salmonella* extends beyond primary tumor sites to execute Step 5 of the cycle (**Figure 1**), the recognition and killing of cancer cells at distant metastatic lesions. For example, a positive feedback loop between the IFN-γ produced by NK cells and the accumulation, activation and cytotoxicity of NK cells due to IFN-γ signaling upon *Salmonella* infection has proven essential to suppress lung metastases derived from mammary, urothelial and colon carcinomas as well as melanoma (16, 39). Similarly, intratumoral *Salmonella* administration prior to tumor excision triggered a systemic, tumor-specific response that requires IFN-γ and CD8^+^ T cells to prevent post-surgical metastatic melanoma dissemination (60). Another approach involving the use of intravenous *Salmonella* inhibited pulmonary melanoma metastases through mechanistic target of rapamycin (mTOR) signaling suppression, which led to the reduction of epithelial-to-mesenchymal transition and diminished cell migration (74). Effective priming and expansion of tumor-specific lymphocytes also enables immune surveillance of distant tissues, contributing to the elimination of circulating tumor cells and the control of metastatic niches. An oral *Salmonella*-based vaccine showed to decrease liver metastases both in number and size when administered 24 hours prior to breast tumor cells injection into the spleen, through the induction of tumor specific T-cells in lymph nodes (75). This capacity to impact metastatic disease distinguishes *Salmonella*-based approaches from therapies that act primarily at the local tumor level and highlights their potential for inducing broad antitumor immunity (**Figure 4**).

Despite these promising effects, primary tumor and metastatic control are not always sustained over time. In some settings, immune cell infiltration diminishes, and antitumor responses wane, suggesting the emergence of regulatory mechanisms that restrain prolonged inflammation (21). These observations raise important questions regarding the balance between immune activation and immune regulation following *Salmonella* treatment and set the stage for exploring the roles of immune memory, trained immunity, and tolerance in shaping long-term outcomes.

## Immune memory, trained immunity, and tolerance: open questions and emerging concepts

One of the most intriguing aspects of attenuated *Salmonella*-based antitumor strategies is their ability to induce systemic immune responses that, in some cases, persist beyond the period of active bacterial presence. Evidence of long-term tumor control and protection against tumor rechallenge in experimental models suggests the induction of immune memory. Supporting this, treatment with an attenuated *Salmonella* Typhi vaccine strain in a murine T-cell lymphoma model resulted in complete tumor eradication in a modest percentage of treated mice, which were subsequently resistant to rechallenge with a lethal dose of the same tumor cells (40). Likewise, intratumor inoculation with *Salmonella* Typhimurium followed by surgical tumor resection resulted in long-lasting antitumor immunity, as evidenced by delayed tumor growth, prolonged survival, and partial resistance to tumor rechallenge (39). Furthermore, in a separate study combining engineered *Salmonella*-mediated delivery of granulocyte-macrophage colony-stimulating factor (GM-CSF) with PD-L1 inhibition, treated mice exhibited a 2-fold increase in memory T cells that resulted in protection against subsequent tumor implantation (76).

Classical adaptive immune memory, mediated by long-lived tumor-specific T and B lymphocytes, likely contributes to sustained antitumor protection following *Salmonella* treatment. The robust activation of CTLs and Th1 cells, together with efficient antigen presentation and inflammatory priming, creates favorable conditions for the generation of memory lymphocyte populations. These cells may provide rapid and effective responses upon tumor recurrence or metastatic dissemination (77). Notably, this robust adaptive protection is tightly coupled with systemic changes; for instance, attenuated *Salmonella* has been shown to induce long-term CD8^+^ T cell-dependent immunity by driving functional modifications in the bone marrow microenvironment, such as the upregulation of interleukin 7 (IL-7) (78).

While memory lymphocytes drive antigen-specific clearance, microbial stimulation simultaneously induces a broader immune reprogramming. Consequently, growing evidence highlights the significant contribution of innate immune memory —also referred to as trained immunity— in sustaining the overall efficacy of microbial-based therapies (79). To evaluate this contribution, it is essential to distinguish trained immunity from other functional states of immune adaptation. Whereas classical adaptive memory relies on epitope-specific lymphocyte clonal expansion, trained innate immunity involves non-antigen-specific reprogramming driven by persistent epigenetic and metabolic modifications in innate immune cells. Crucially, unlike cellular priming, where activation remains elevated, trained cells return to a basal transcriptional state after initial stimulus clearance, yet mount a heightened response upon restimulation. Conversely, innate tolerance is a refractory state marked by epigenetic silencing (e.g., following high-dose LPS exposure) that prevents inflammatory gene expression, while immune exhaustion reflects the progressive loss of T cell effector function during chronic stimulation (80). In the context of *Salmonella*-based cancer therapy, trained innate responses could contribute to heightened antitumor surveillance and sustained inflammatory competence. A recent study has shown that a single dose of therapeutic *Salmonella* provides extensive and persistent protection against different cancer derived-metastases by inducing trained immunity in NK cells, characterized by enhanced capacity to release IFN-γ upon secondary stimulation and increased cytotoxicity toward cancer cells (81). Another study also showed that attenuated *Salmonella* induces a robust cytokine response in bone marrow cells upon a second stimulus, and when intraperitoneally injected one week previous to tumor implantation, also enhanced antitumor responsiveness, as evidenced by a slowed tumor growth and prolonged survival (38).

Conversely, repeated or prolonged exposure to bacterial components may also induce immune tolerance, particularly within the innate immune compartment. Endotoxin tolerance and related regulatory states are associated with diminished pro-inflammatory responses upon restimulation and may represent a counter-regulatory mechanism that limits excessive inflammation (82–84). *In vitro* studies have shown that LPS stimulation via TLR4 enhances the suppressive activity of Tregs, increasing their capacity to inhibit conventional T cell proliferation (54). In line with this concept, *in vitro* stimulation of human monocytes and murine bone marrow–derived myeloid-enriched cells with attenuated *Salmonella* results in reduced cytokine production, consistent with an immune paralysis–like state (38). Together, these findings support the notion that *Salmonella*-based immunotherapies may simultaneously engage trained and tolerogenic programs, potentially contributing to the transient nature of antitumor efficacy observed in most experimental models.

The coexistence of immune activation, trained immunity, and immune tolerance underscores the dynamic and context-dependent nature of *Salmonella*-induced immune modulation. The balance among these states likely depends on multiple factors, including bacterial dose, route and timing of administration, tumor type, and host immune status, and these programs are not mutually exclusive but instead may operate sequentially or in parallel to shape therapeutic outcomes. Dissecting how attenuated *Salmonella* navigates this immunological spectrum remains a critical challenge for the field. Determining whether durable antitumor effects are primarily driven by adaptive immune memory, trained innate responses, or their combination will be essential for the rational design of improved *Salmonella*-based immunotherapies. On the other hand, identifying the conditions under which tolerogenic programs emerge may inform strategies to sustain efficacy, including optimized dosing regimens or combination approaches with immune checkpoint inhibitors or metabolic modulators. Together, these observations support a model in which attenuated *Salmonella* initiates a layered immune response, where early innate activation and inflammatory priming can give rise to trained immunity, subsequently facilitating the development of durable adaptive immune memory. In parallel, sustained or repeated stimulation may engage regulatory and tolerogenic programs that limit excessive inflammation but also constrain long-term efficacy. Thus, antitumor outcomes likely emerge from the dynamic interplay and temporal convergence of these processes, rather than from any single mechanism in isolation.

## Concluding remarks and future perspectives

Attenuated *Salmonella* represents a unique immunotherapeutic platform capable of modulating multiple steps of the cancer–immunity cycle. Beyond its capacity to directly induce tumor cell death, *Salmonella* orchestrates a coordinated immune response that integrates innate activation, efficient antigen presentation, adaptive lymphocyte priming, tumor infiltration, and systemic immune surveillance (**Table 1**). Specifically, across the cycle, *Salmonella* initiates Step 1 by inducing direct tumor cell death, facilitates Step 2 through dendritic cell maturation and antigen presentation, drives Step 3 by activating adaptive immune responses, promotes Step 4 by driving immune cell trafficking and infiltration back into the primary tumor, and enforces Step 5 by controlling metastatic disease (**Figure 1**). This multifaceted mode of action distinguishes *Salmonella*-based approaches from therapies that target isolated immune pathways and underscores their potential as immune modulators rather than solely cytotoxic agents or delivery vectors. However, key unresolved questions remain regarding how to strategically leverage and amplify this multi-step cycle to maximize its endurance, particularly in defining how bacterial parameters can be tailored to extend effector signaling, prevent premature immune exhaustion, and sustain a self-reinforcing feedback loop that ensures long-term antitumor durability.

**Table 1:** Modulation of the cancer–immunity cycle by *Salmonella*.

Cancer-immunity cycle step		*Salmonella-*mediated mechanism		Strains	Model	References
1	Cancer cell death	Invasion and direct tumor cell killing		*Salmonella* Typhimurium (ST): VNP20009, YB1, YS8211, YS1629, YS721, YS7211, YS7212, YS7213, A1	*In vivo*: B16F10 murine melanoma, 4T1 murine breast cancer, CT26 murine colon cancer, LOX human melanoma, DLD-1 human colon carcinoma, PC-3 human prostate cancer.	15–19
		Induction of tumor stress		ST: SL1344, SopB-, SL3261	*In vitro*: MDAMC human breast carcinoma epithelial cells, HeLA human epithelial cell line. *In vivo*: C57BL/6 induced colitis-associated colorectal cancer model.	27, 28
		Immunogenic cell death		ST: VNP20009; VNP-tdT, LVR01	*In vitro*: B16F1 murine melanoma cells. *In vivo*: B16F10 murine melanoma, A20 murine lymphoma.	11, 29, 30
		Release of tumor antigens and DAMPs		ST: LVR01, SL1344, BJ66	*In vitro*: B16F1 murine melanoma cells. *Ex vivo*: BMDMs bone marrow-derived macrophages.	30, 32
		Remodeling of TME	Proinflammatory	ST: VNP20009, LVR01; *Salmonella* Choleraesuis (SC): ATCC 15480	*In vitro*: RAW 264.7 murine monocyte-macrophage cell line. *Ex vivo*: BMDMs bone marrow-derived macrophages, BMDCs bone marrow-derived dendritic cells, PBMCs human peripheral blood mononuclear cells. *In vivo*: B16F1, B16F10 and K1735 murine melanoma, LLC murine lung carcinoma, A20 murine lymphoma.	34–36, 30, 37, 38
			Antitumoral	ST: LVR01, BRD509E; *Salmonella* Typhi: CVD915; SC: ATCC 15480	*In vivo*: B16F1 and B16F10 murine melanoma, EL4 murine metastatic T-cell lymphoma, A20 murine lymphoma, MC38 murine colon adenocarcinoma, LL2 murine lung carcinoma, 4T1 murine breast cancer.	39, 40, 11, 44–47
2	Cancer antigen presentation and priming	Recruitment and activation of DCs		ST: VNP20009; VNP-tdT	*In vivo*: B16F10 murine melanoma.	29
		Strong maturation signals to DCs		ST: 14028r, X4550	*Ex vivo*: splenic mouse dendritic cells, BMDMs bone marrow-derived macrophages, BMDCs bone marrow-derived dendritic cells.	48, 49
		Cross-presentation	Increased Cx43 and reduced IDO expression	ST: SL3261; SC: ATCC 15480	*In vitro*: B16F10 murine melanoma cells, 4T1 murine breast cancer cells. *In vivo*: B16F10 murine melanoma.	52, 53
		Priming of tumor-specific B and T cells				
			TDLN lymphocytes decreased CTLA-4 and PD-1 expression	ST: LVR01; *S.* Typhi: CVD915	*In vivo*: B16F1 murine melanoma, EL4 murine metastatic T-cell lymphoma.	39, 40
			Increased splenic CD4+T cells	ST: BRD509E	*In vivo*: MC38 murine colon adenocarcinoma.	44
3	Activation of adaptive immune responses	Induction of systemic antitumor immunity	CD8+ CTLs	ST: BRD509, SL1344, LVR01; SC: ATCC 15480	*In vivo*: CT26 murine colon cancer, B16F1 and B16F10 murine melanoma, LL2 murine lung carcinoma, A20 murine lymphoma.	22, 37, 39, 45, 55
			CD4+ Th1	ST: LVR01; Sc: ATCC 15480; *S*. Typhi: CVD915	*In vivo*: B16F1 and B16F10 murine melanoma, LL2 murine lung carcinoma, A20 murine lymphoma, LM3 murine mammary adenocarcinoma.	45, 55, 61, 64
			B cells, Antibodies	ST: LVR01, YB1; S. Typhi: CVD915	*In vivo*: B16F1 murine melanoma, EL4 murine metastatic T-cell lymphoma, A20 murine lymphoma, MB49 murine bladder cancer.	11, 23, 40, 43
4	Lymphocyte trafficking and tumor infiltration	Inflammatory remodeling of the tumor microenvironment promoting recruitment of lymphocytes and innate immune effectors		ST: LVR01, SL3261, VNP20009	*In vivo*: B16F1 murine melanoma, A20 murine lymphoma, CT26 murine colon cancer, C57BL/6 and C57BL/6ICAM-1^−^/^−^ mice.	11, 39, 70, 71
5	Elimination of metastatic tumor cells	Systemic immune activation and potential bacterial colonization of metastatic niches contributing to metastatic control		ST: YB1, LVR01; S. Typhi: CVD915	*In vivo*: 4T1 murine breast cancer, B16F10 murine melanoma, LM3 murine mammary adenocarcinoma.	16, 39, 60, 61

CTL, cytotoxic T cell; CTLA-4, cytotoxic T-lymphocyte antigen 4; Cx43, Connexin 43; DC, dendritic cell; IDO, indoleamine 2, 3-dioxygenase; PD-1, programmed cell death protein 1; SC, Salmonella enterica Choleraesuis; ST, Salmonella enterica Typhimurium; TDLN, tumor-draining lymph node.

Importantly, while preclinical models demonstrate that *Salmonella* therapy can induce durable protection capable of rejecting secondary tumor re-challenges, translating this into long-term adaptive memory in clinical settings remains a major gap. Current evidence suggests that *Salmonella* shifts the TME toward a pro-inflammatory state, facilitating the generation of tumor-specific memory T cells. Nevertheless, the antitumor effects of attenuated *Salmonella* are not uniformly durable, highlighting the complex balance between immune activation, regulation, and exhaustion. The emerging roles of adaptive immune memory, trained immunity, and immune tolerance provide a conceptual framework to understand both sustained and transient therapeutic outcomes. Elucidating how these immune programs are differentially engaged in distinct experimental and clinical contexts will be critical for improving the efficacy and predictability of *Salmonella*-based strategies.

While VNP20009 historically pioneered intravenous bacterial immunotherapy in humans (85), subsequent clinical trials have evaluated other attenuated strains, such as the oral IL-2–expressing strain χ4550 (Saltikva) (86–88) and more recently the L-methioninase–producing SGN1 strain (ClinicalTrials.gov: NCT05103345, NCT05038150), expanding the translational landscape of *Salmonella*-based oncotherapies. Despite proving to be safe, to date none of them has achieved complete clinical efficacy. This may be explained by the delicate balance required between safety and potency: to prevent life-threatening bacteremia, endotoxic shock and severe systemic toxicity, bacterial platforms are often over-attenuated, which inadvertently dampens their intrinsic immunogenicity and therapeutic persistence within the tumor. To overcome these translational hurdles, alternative strategies are being actively explored. These include surface engineering approaches to enhance tumor-specific homing—such as displaying RGD homing peptides on outer membrane proteins (OmpA) to selectively target tumor vasculature and integrin-overexpressing cells (89), the engineering of obligate anaerobic strains—such as the abovementioned *Salmonella* YB1—that strictly replicate within hypoxic necrotic tumor cores without persisting in normoxic systemic tissues (90), or the use of non-replicating bacterial derivatives like outer membrane vesicles (OMVs), which deliver potent pathogen-associated molecular patterns and payloads while eliminating the risks of active infection (91). Of note, preexisting immunity against the bacterial agent must be taken into account. To surpass this limitation, a strategy combining lipid A and flagellin modifications was developed to enhance immunostimulatory capacity, yielding the engineered strain SF200 (92). Furthermore, strategies such as OMVs, localized strains with restricted systemic distribution, or LPS modification may mitigate endotoxin tolerance. By dampening excessive systemic LPS exposure, these approaches attenuate the initial hyper-inflammatory burst and limit circulating bacterial loads, thereby preserving systemic immune responsiveness while maintaining potent local antitumor activity.

Future studies should focus on defining the parameters that govern the immunological balance, including bacterial dose, timing, and route of administration, as well as tumor-specific and host-related factors. In parallel, combination approaches integrating attenuated *Salmonella* with immune checkpoint blockade, metabolic interventions, or other immunomodulatory agents may help to reinforce antitumor immunity while preventing the emergence of tolerogenic or exhausted states.
